# Anti-Inflammatory Activity of *Sanghuangporus sanghuang* Mycelium

**DOI:** 10.3390/ijms18020347

**Published:** 2017-02-07

**Authors:** Wang-Ching Lin, Jeng-Shyan Deng, Shyh-Shyun Huang, Sheng-Hua Wu, Chin-Chu Chen, Wan-Rong Lin, Hui-Yi Lin, Guan-Jhong Huang

**Affiliations:** 1 School of Pharmacy, China Medical University, Taichung 404, Taiwan; linwc0913@gmail.com (W.-C.L.); sshuang@mail.cmu.edu.tw (S.-S.H.); 2 Department of Health and Nutrition Biotechnology, Asia University, Taichung 413, Taiwan; dengjs@asia.edu.tw; 3 Department of Biology, National Museum of Natural Science, Taichung 404, Taiwan; shwu@mail.nmns.edu.tw; 4 Grape King Biotechnology Center, Chung-Li City 320, Taiwan; gkbioeng@grapeking.com.tw; 5 Department of Chinese Pharmaceutical Sciences and Chinese Medicine Resources, College of Pharmacy, China Medical University, Taichung 404, Taiwan; linwr0627@gmail.com

**Keywords:** acute lung injury, mycelium of *Sanghuangporus sanghuang*, lipopolysaccharide, KAP1/Nrf2 pathway, PI3K/Akt/mTOR pathways, HO-1, HNGB

## Abstract

Acute lung injury (ALI) is characterized by inflammation of the lung tissue and oxidative injury caused by excessive accumulation of reactive oxygen species. Studies have suggested that anti-inflammatory or antioxidant agents could be used for the treatment of ALI with a good outcome. Therefore, our study aimed to test whether the mycelium extract of *Sanghuangporus sanghuang* (SS-1), believed to exhibit antioxidant and anti-inflammatory properties, could be used against the excessive inflammatory response associated with lipopolysaccharides (LPS)-induced ALI in mice and to investigate its possible mechanism of action. The experimental results showed that the administration of SS-1 could inhibit LPS-induced inflammation. SS-1 could reduce the number of inflammatory cells, inhibit myeloperoxidase (MPO) activity, regulate the TLR4/PI3K/Akt/mTOR pathway and the signal transduction of NF-κB and MAPK pathways in the lung tissue, and inhibit high mobility group box-1 protein 1 (HNGB1) activity in BALF. In addition, SS-1 could affect the synthesis of antioxidant enzymes Heme oxygenase 1 (HO-1) and Thioredoxin-1 (Trx-1) in the lung tissue and regulate signal transduction in the KRAB-associated protein-1 (KAP1)/nuclear factor erythroid-2-related factor Nrf2/Kelch Like ECH associated Protein 1 (Keap1) pathway. Histological results showed that administration of SS-1 prior to induction could inhibit the large-scale LPS-induced neutrophil infiltration of the lung tissue. Therefore, based on all experimental results, we propose that SS-1 exhibits a protective effect against LPS-induced ALI in mice. The mycelium of *S. sanghuang* can potentially be used for the treatment or prevention of inflammation-related diseases.

## 1. Introduction

Acute Lung Injury (ALI) is a condition where lung tissue is injured by excessive nonspecific acute inflammatory response in the lungs [1]. In addition, ALI can lead to acute respiratory failure, and even acute respiratory distress syndrome (ARDS), the most severe manifestation of ALI [2]. ALI and ARDS are common clinical complications in critically ill patients and can cause septic shock and metabolic acidosis resulting in a very high mortality [3]. The main physiopathological mechanism of ALI is the exacerbation of the inflammatory response [2,3]. Excessive cytokine synthesis stimulates the response of inflammatory cells, leading to the disruption of alveolar epithelial cells and pulmonary microvascular endothelial cells. Meanwhile, the immune cells (macrophages, monocytes and neutrophils) synthesize numerous pro-inflammatory mediators from the phospholipids of the cell membrane to enhance the inflammatory response [1,2]. The lung injury caused by excessive inflammation alters vascular permeability, which then causes alveolar infiltration and destroys the normal diffusing capacity of the lung, thereby resulting in hypoxia [1,2,3].

Macrophages and neutrophils play important roles in the pathogenesis of ALI. Previously, only the role of neutrophils was considered significant, and that of macrophages was found to be non-negligible [4]. In addition to synthesizing active lipid metabolites and oxygen free radicals, macrophages can also produce tumor necrosis factor (TNF) and interleukin (IL), as well as activate the neutrophils. Macrophages and neutrophils can interact with and activate each other, leading to the exacerbation of the lung injury [5].

Studies have found that high mobility group protein (HMGB1) is translocated from the nucleus to the cytoplasm during cell injury and then actively secreted from immune cells or directly released from necrotic cells [6]. Extracellular HMGB1 binds to the cell surface receptors (RAGE and TLR4) and triggers the downstream NF-κB pathway via MyD88 protein, thereby promoting the secretion of pro-inflammatory cytokines and chemokines to regulate the inflammatory response of cells [7].

The Phosphoinositide 3-kinase (PI3K)/Akt signaling pathway affects cell survival and is associated with LPS-induced signal transduction [8]. Akt regulates LPS-induced NF-κB activation and promotes NF-κB phosphorylation via IκB kinase (IKK), thus enhancing the transcription ability of NF-κB [9]. Recent studies indicate that Akt also regulates mechanistic target of rapamycin (mTOR) activity, when mTOR binds to and phosphorylates IKK after being phosphorylated by Akt, thereby promoting the NF-κB activation [10]. On the contrary, the inhibition of mTOR reduces LPS-induced synthesis of pro-inflammatory cytokines and NF-κB phosphorylation, indicating that the PI3K/Akt/mTOR/IKK signal transduction pathways greatly affect the activation of NF-κB [8,9,10,11].

Previous studies have shown that Keap1 is a negative regulator of Nrf2. Under conditions of oxidative stress, Keap1 releases Nrf2, which translocates into the nucleus to activate the transcription of various downstream antioxidant genes, such as HO-1 and Trx-1, enabling cells to metabolize the free radicals in order to survive oxidative and electrophilic stress [12,13]. Current studies suggest that the PI3K and MAPK pathways are mainly involved in signal transduction, nuclear factor erythroid-2-related factor (Nrf2)/antioxidant responsive element (ARE) activation, and downstream gene expression of phase II detoxification enzymes and antioxidant enzymes [12,13,14,15]. On the contrary, KRAB-associated protein 1 (KAP1, also known as tripartite motif-containing protein 28, TRIM28), a Trim family protein, inhibits transcription in numerous transcriptional regulatory complexes; however, the inhibitory mechanism of KAP1 is still unclear [16]. Previous studies have suggested that KAP1 serves as a positive regulator of Nrf2, which enters the nucleus to initiate transcription and affects the release of antioxidant enzymes [17].

Pathological analysis of tissue after ALI reveals infiltration by neutrophils and transformation of mononuclear cells into pro-inflammatory macrophages that continue to proliferate, thereby altering the immune cell morphology in the tissue [18]. Therefore, the three key factors in alleviating the inflammatory response include the reduction of immune cell infiltration, the inhibition of excessive inflammatory response, and the removal of immune cells that promote inflammatory response in the tissue [18,19,20].

*Sanghuangporus sanghuang*, a precious medicinal fungus, has been in circulation in China, Japan, and South Korea for thousands of years [21,22]. The species remained unknown until identified by Wu et al. in 2012 as *Inonotus sanghuang* [23]. Thereafter, it was reclassified as a new genus, *Sanghuangporus*, in 2015 and its scientific name was changed to *S. sanghuang* [24]. *S. sanghuang* is distributed throughout Taiwan, Japan, South Korea, and China. It only grows on mulberry trees in the wild with an extremely scarce yield [25,26]. To satisfy the demand, consumers rely on extensive artificial cultivation; however, there are no studies investigating the anti-inflammatory and pharmacological properties of the mycelium of *S. sanghuang* grown on wild mulberry trees, or its use for the treatment of LPS-induced ALI.

Therefore, our study aimed to determine whether the mycelium of *S. sanghuang* exhibits anti-inflammatory properties and evaluate its anti-inflammatory effectiveness via a mouse model with endotoxin-induced ALI, as well as investigate a possible mechanism of action.

## 2. Results

### 2.1. Cytotoxicity and NO Inhibition

**Figure 1:**
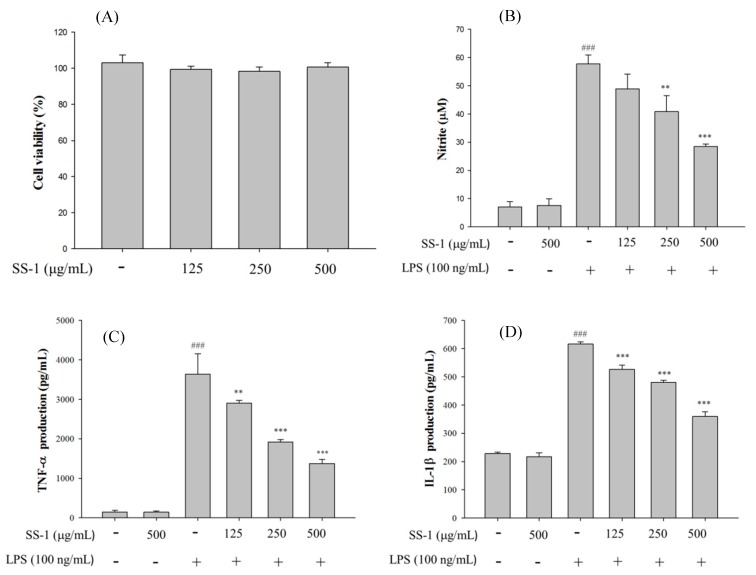

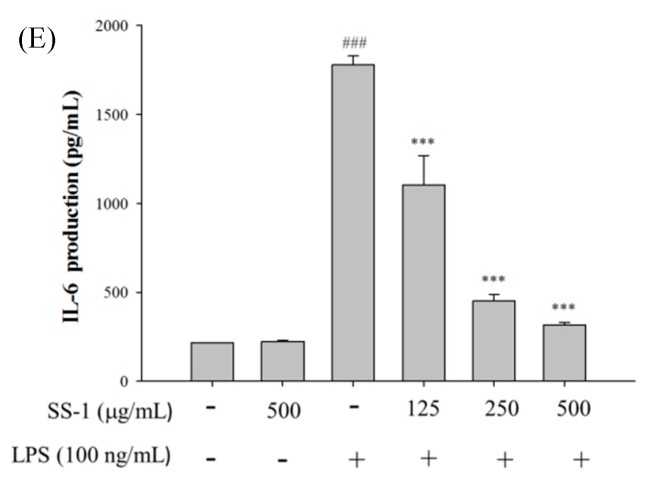
SS-1 inhibited lipopolysaccharide (LPS)-induced cell inflammation in RAW264.7 cells. Cytotoxicity (**A**) of SS-1 in LPS-stimulated RAW264.7 cells. Cells were treated with SS-1 at 125, 250 and 500 μg/mL for 24 h, and cell viability was assayed by the 3-(4,5-Dimethylthiazol-2-yl)-2,5-diphenyltetrazolium bromide MTT assay. NO (**B**); TNF-α (**C**); IL-1β (**D**); and IL-6 (**E**) production in LPS-stimulated RAW264.7 cells. Cells were incubated with or without LPS (100 ng/mL) in the presence of various doses (125, 250 and 500 μg/mL) of SS-1 for 24 h. Data are expressed as the means ± SD of three independent experiments; ^###^ compared with sample of control group (one-way ANOVA followed by Scheffe’s multiple range tests); ** *p* < 0.01, and *** *p* < 0.001, were compared with LPS-alone group.

As shown in Figure 1, different doses of SS-1 do not affect the survival of macrophages and the administration of SS-1 at non-cytotoxic doses significantly reduced LPS-induced NO production and the concentration of pro-inflammatory cytokines in cells. SS-1 reduced the NO, TNF-α, IL-1β, and IL-6 production of activated macrophages with an IC_50_ value of 491.5, 262.9, 606.2, and 166.2 μg/mL, respectively.

### 2.2. Effects of SS-1 on LPS-Induced iNOS, COX-2, NF-κB, MAPK and TLR4/PI3K/Akt/mTOR/IKKβ Protein Expressions in Macrophages

**Figure 2:**
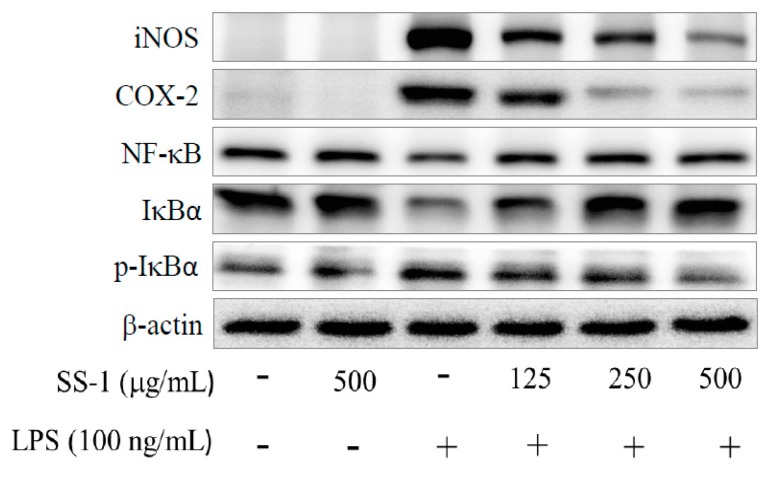

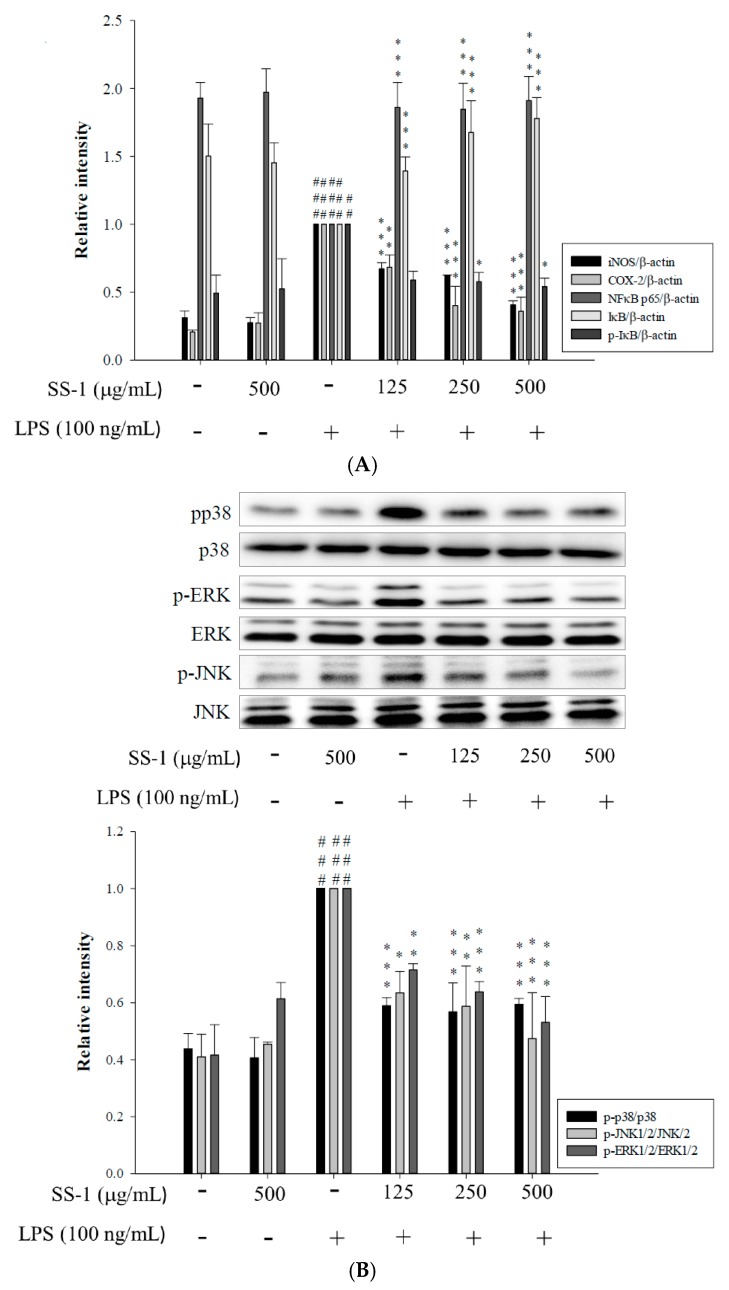
Effects of SS-1 on iNOS, COX-2, IκBα, p-IκBα, and NF-κB protein expression (**A**); and MAPK phosphorylation (**B**) in LPS-induced RAW264.7 cells. Cells were incubated with or without LPS (100 ng/mL) in the presence of various concentrations (125, 250 and 500 μg/mL) of SS-1 for 24 h. The data were presented as mean ± SD for the three different experiments performed in triplicate; ^##^ *p* < 0.01, and ^###^ *p* < 0.001 were compared with sample of control group (one-way ANOVA followed by Scheffe’s multiple range tests); * *p* < 0.05, ** *p* < 0.01 and *** *p* < 0.001 were compared with LPS-alone group.

**Figure 3:**
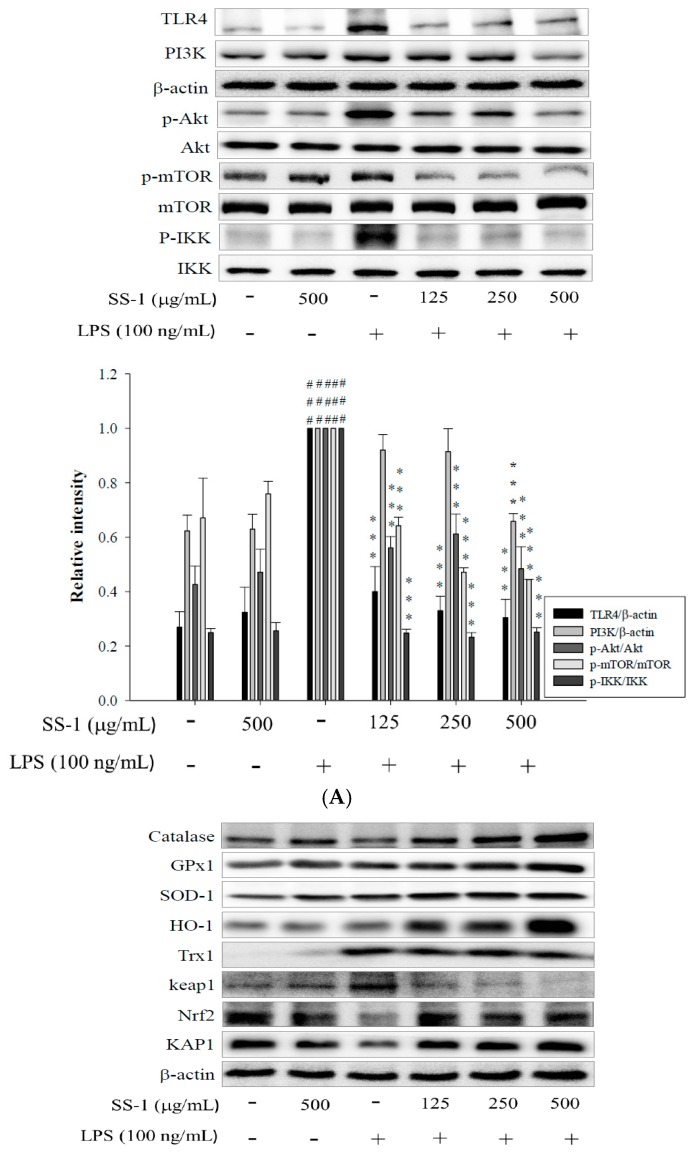

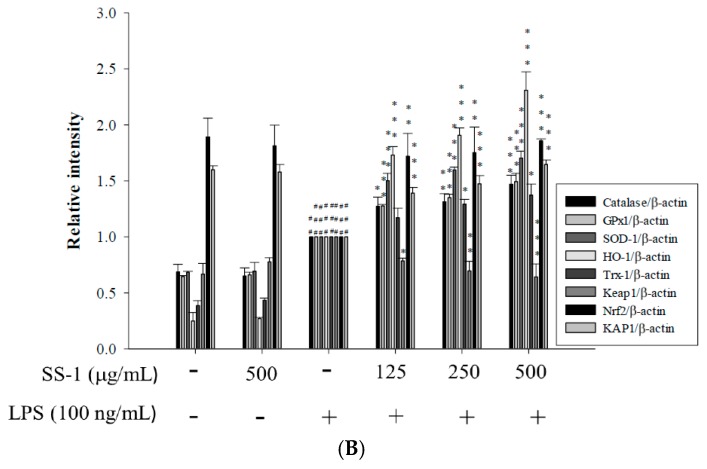
Effects of SS-1 on TLR4/PI3K/Akt/mTOR/IKK protein expression (**A**); and anti-oxidative enzymes, HO-1, Trx-1, and Nrf2/KAP1 protein expression (**B**) in LPS-induced RAW264.7 cells. Cells were incubated with or without LPS (100 ng/mL) in the presence of various concentrations (125, 250 and 500 μg/mL) of SS-1 for 24 h. The data were presented as mean ± SD for the three different experiments performed in triplicate; ^##^ *p* < 0.01, and ^###^ *p* < 0.001 were compared with sample of control group (one-way ANOVA followed by Scheffe’s multiple range tests); ** *p* < 0.01 and *** *p* < 0.001 were compared with LPS-alone group.

Meanwhile, we also found that SS-1 could significantly reduce the expression of iNOS and COX-2, as shown in Figure 2. Our results show that incubation of RAW264.7 cells with SS-1 enhances the cytoplasmic expression levels of NF-κB p65 and IκBα proteins, while reducing the cytoplasmic expression level of p-IκBα protein. At the same time, SS-1 significantly regulates the phosphorylation levels of ERK 1/2, JNK 1, and p38 proteins in the cells. Compared with the LPS group, the SS-1 administered group showed a significant reduction in the activity of the TLR4/PI3K/Akt/mTOR/IKKβ signaling pathway, as shown in Figure 3A.

### 2.3. Effects of SS-1 on LPS-Induced Antioxidative Enzymes and HO-1, Trx-1, KAP1/Nrf2 Protein Expressions in Macrophage

As shown in Figure 3B, we found that administration of SS-1 could significantly upregulate the protein expression levels of glutathione peroxidase (GPX), superoxide dismutase (SOD-1), catalase (CAT), heme oxygenase-1 (HO-1), and thioredoxin-1 (Trx-1) antioxidant enzymes in cells, as well as significantly affect the expression levels of KAP1/Nrf2 proteins.

### 2.4. Effects of SS-1 on LPS-Mediated Lung Histopathologic Changes

**Figure 4:**
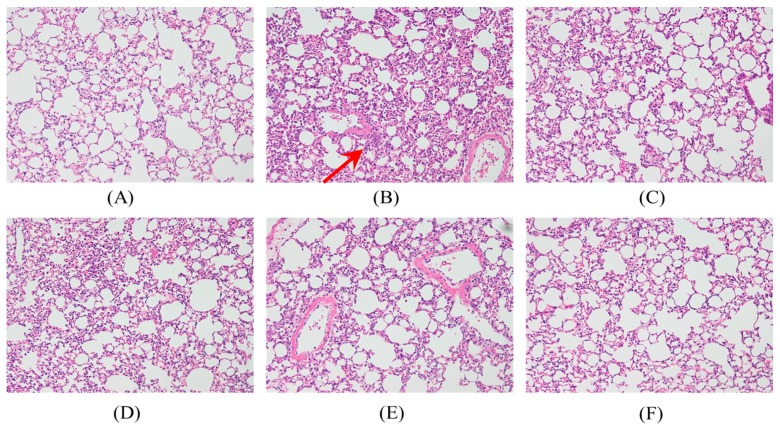

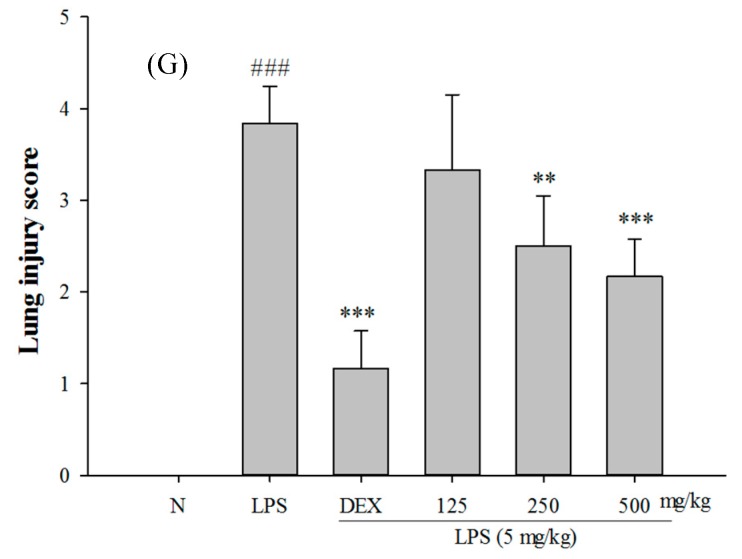
SS-1 attenuated pulmonary inflammation in vivo. Six hours after LPS injection with or without SS-1 pretreatments, mice were exsanguinated and their left lower lungs were fixed. Then, tissue sections were stained with hematoxylin and eosin (H&E): (**A**) Control; (**B**) LPS; (**C**) LPS + Dex; (**D**) LPS + SS-1-L; (**E**) LPS + SS-1-M; (**F**) LPS + SS-1-H. The figure demonstrates a representative view (×400) from each group; each bar represents the mean ± SD of six mice. (**G**) Severity of lung injury was analyzed by the lung injury scoring system. Each value represents as mean ± SD of six mice; ^###^ compared with sample of control group; ** *p* < 0.01, and *** *p* < 0.001 were compared with LPS-alone group. The infiltrating neutrophils were more abundant in (**B**) LPS group as shown by arrows.

In Figure 4, a histological section of lung tissue shows significant thickening of edema and leukocyte infiltration in the alveoli and interstitial space of the tissue in the samples induced with LPS. The administration of SS-1 and Dex prior to LPS induction could alleviate edema and leukocyte infiltration in the alveoli and interstitial space.

### 2.5. SS-1 Attenuates Pulmonary Edema and Reduces Cellular Counts and Proteins in BALF in LPS-Induced ALI Mice

**Figure 5:**
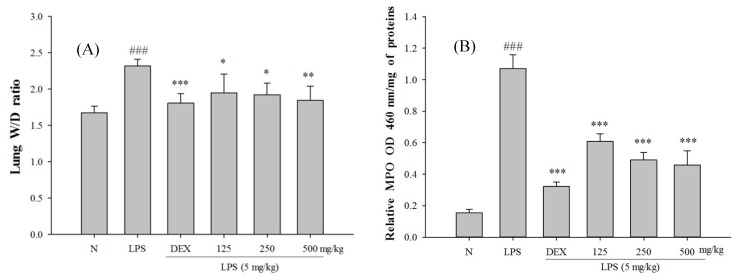

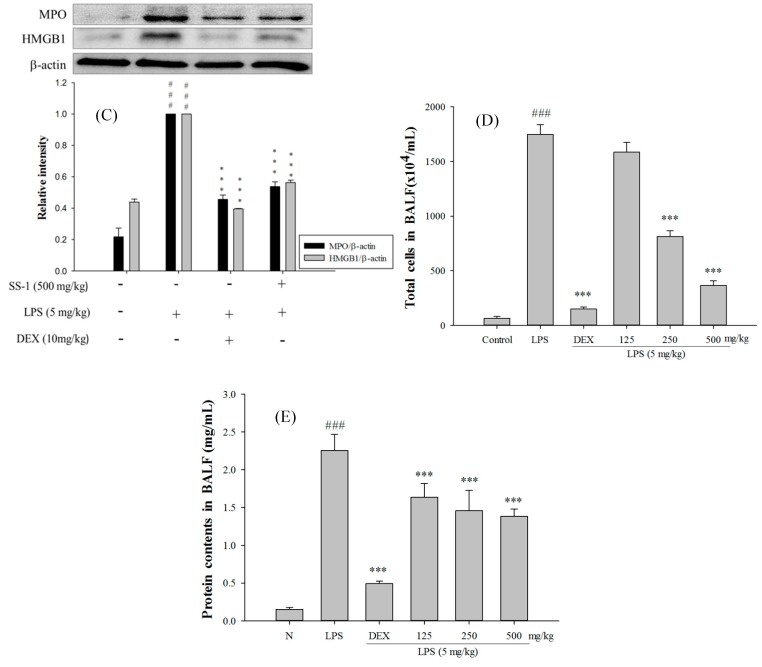
SS-1 improved pulmonary edema (**A**); Myeloperoxidase (MPO) activity (**B**); MPO and HMGB1 protein level (**C**) in vivo; and reduced cellular counts (**D**); and total protein (**E**) in BALF. Six hours after LPS injection with or without SS-1 pretreatments, mice were sacrificed and their lungs were lavaged. The right lower lungs were used to assess wet to dry (W/D) ratio of lung. Cells in the BALF were collected and cytospin preparations were made. Total cells and total proteins in BALF were analyzed. Data represent mean ± SD of six mice; ^###^ compared with sample of control group (one-way ANOVA followed by Scheffe’s multiple range tests); * *p* < 0.05, ** *p* < 0.01, and *** *p* < 0.001, were compared with LPS-alone group.

The SS-1-treated group exhibited significantly reduced wet/dry weight ratio of lung tissue, MPO activity, and total cell count and protein concentration in bronchoalveolar lavage fluid (BALF) than the LPS-induced group, as shown in Figure 5. Furthermore, in comparison with the LPS-induced group, the SS-1-treated group had a significantly lower HMGB1 protein expression level in the lung tissue.

### 2.6. Effect of SS-1 on BALF Cytokine Levels and iNOS, COX-2 Protein Expressions in LPS-Induced ALI Mice

**Figure 6:**
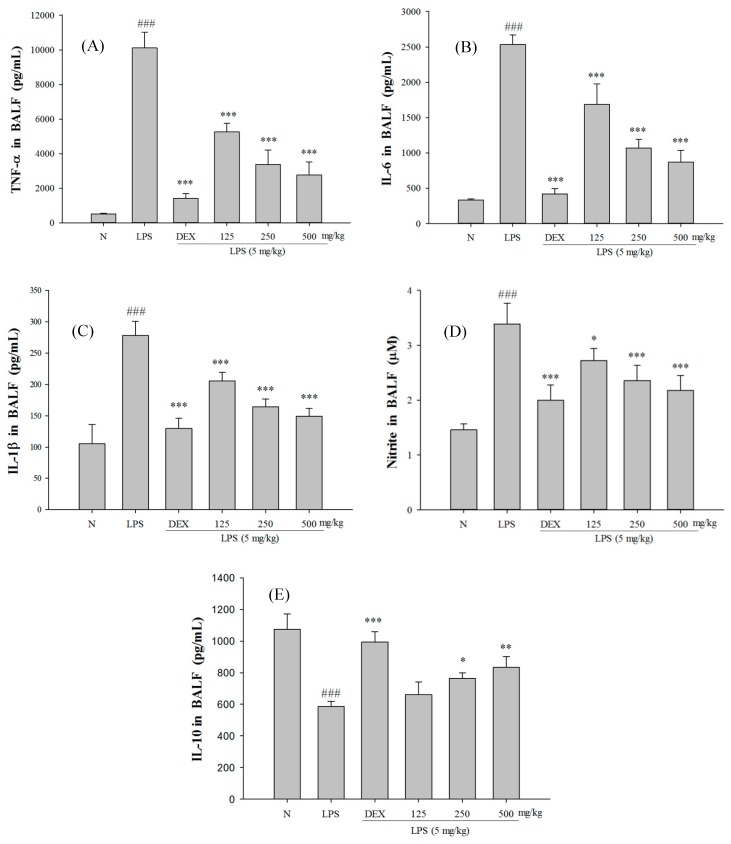
SS-1 down regulated: TNF-α (**A**); IL-6 (**B**); IL-1β (**C**); and NO (**D**); and increased IL-10 (**E**) in BALF. Six hours after LPS injection with or without SS pre-treatments, mice were sacrificed, their lungs were lavaged and the BALF were collected. TNF-α, IL-6, IL-1β, NO and IL-10 were detected by ELISA. Data represent mean ± SD of six mice; ^###^ compared with sample of control group (one-way ANOVA followed by Scheffe’s multiple range tests); * *p* < 0.05 ** *p* < 0.01, and *** *p* < 0.001, were compared with LPS-alone group.

Excessive concentrations of NO and pro-inflammatory cytokines in the body play a key role in the pathogenesis of LPS-induced ALI. As shown in Figure 6, the LPS-induced group has significantly higher concentrations of TNF-α, IL-1β, IL-6, and NO in BALF than the control group. On the contrary, the group that was administered SS-1 showed significantly lower concentrations of TNF-α, IL-1β, IL-6, and NO in BALF than the LPS-induced group. In addition, the SS-1 treated group had a significantly higher IL-10 concentration in BALF.

**Figure 7:**
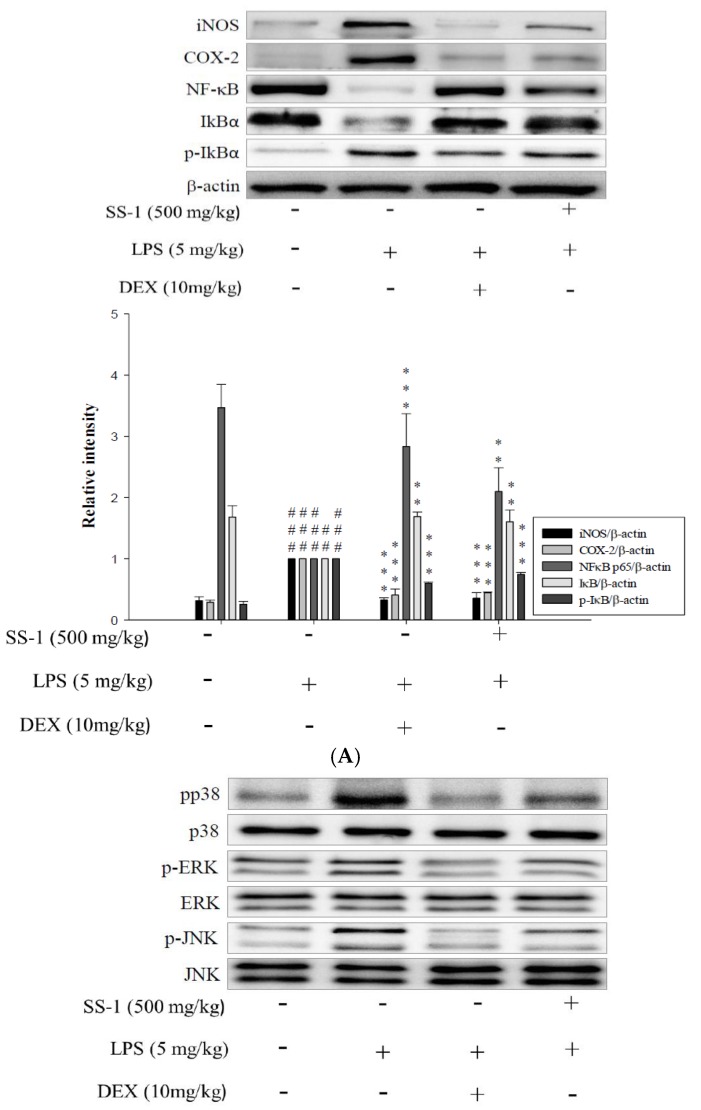

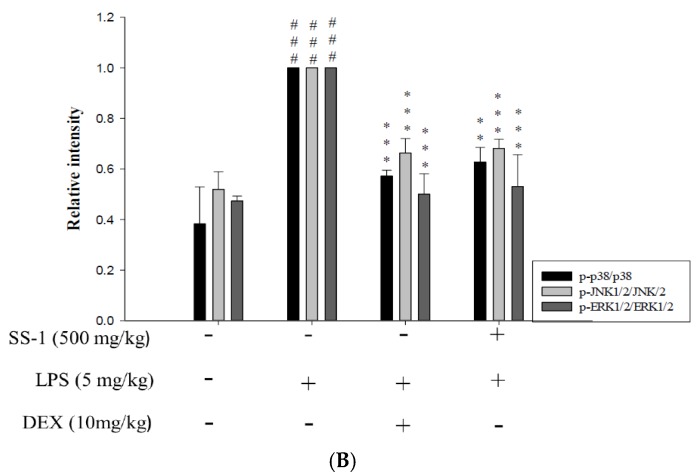
Effects of SS-1 on LPS-induced iNOs, COX-2, IκB-α, and NF-κB protein expression in lung (**A**); and MAPK phosphorylation (**B**) expression in ALI mice. Mice were pretreated with different concentrations of SS for 1 h and stimulated with LPS. Western blotting using specific antibodies was used for the detection of iNOs, COX-2, IκB-α phosphorylated, NF-κB nuclear and cytosol, and total forms of three MAPK molecules, ERK, p38, and JNK. Data represent mean ± SD of six mice; ^##^ *p* < 0.01, and ^###^ *p* < 0.001 were compared with sample of control group (one-way ANOVA followed by Scheffe’s multiple range tests); ** *p* < 0.01, and *** *p* < 0.001, were compared with LPS-alone group.

The expression levels of iNOS and COX-2 proteins were analyzed using a Western blot. As shown in Figure 7A, the LPS-induced group showed significantly higher expression levels of iNOS and COX-2 proteins than the control group, whereas the SS-1-treated group had significantly lower expression levels of iNOS and COX-2 proteins in the lung tissue than the LPS-induced group. 

### 2.7. Effects of SS-1 on NF-κB and MAPK Activation in LPS Induced ALI Mice

LPS induction activates the NF-κB and MAPK pathways in the lungs of mice with ALI. As shown in Figure 7A, the LPS-induced group had lower cytoplasmic expression levels of NF-κB p65 and IκBα proteins, and higher cytoplasmic expression levels of p-IκBα, indicating that induction with LPS could cause the activation and nuclear translocation of NF-κB p65. The administration of SS-1 prior to induction increased the cytoplasmic expression levels of NF-κB p65 and IκBα, while reducing the cytoplasmic p-IκBα protein expression levels. These results suggest that SS-1-treated group exhibits reduced the nuclear translocation of NF-κB p65 at significant levels when compared to that of the LPS-induced group. Additionally, the SS-1-treated group exhibited significantly lower phosphorylation levels of ERK 1/2, JNK 1 and p38 proteins in lung tissue than the LPS-induced group, as shown in Figure 7B.

### 2.8. Effect of SS-1 on PI3K/Akt/mTOR /IKKβ Pathway Activation in LPS Induced ALI Mice

**Figure 8:**
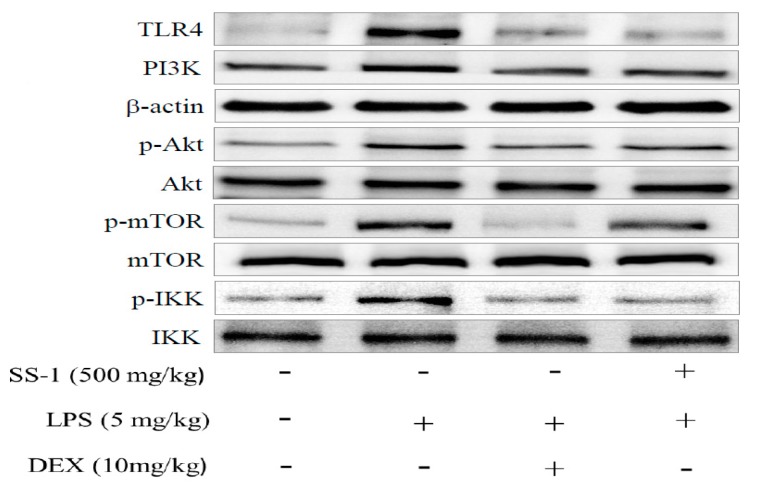

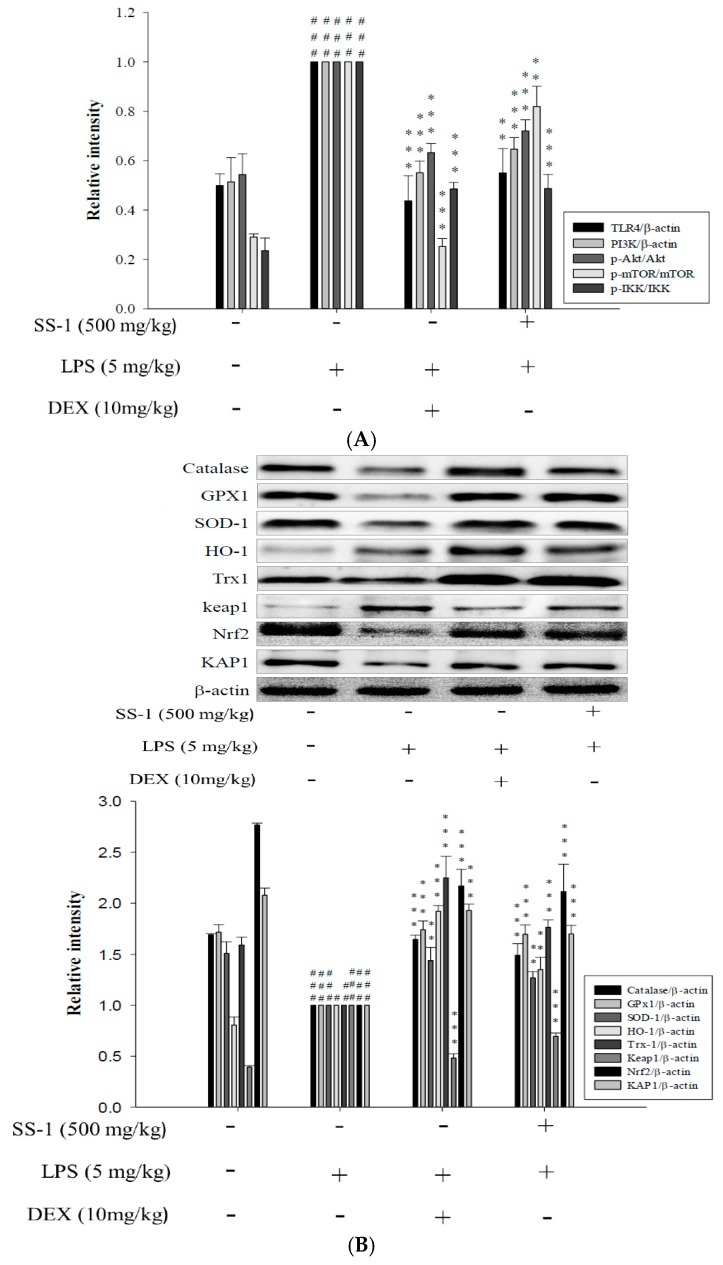
Effects of SS-1 on LPS-induced TLR4, PI3K, AKT, mTOR, and IKK protein expression (**A**); and antioxidative enzymes and HO-1, Trx-1, Nrf2/KAP1 protein expression (**B**) in lung in ALI mice. Mice were pretreated with different concentrations of SS for 1 h and stimulated with LPS. The Western blotting using specific antibodies was used for the detection of TLR4, PI3K, AKT, mTOR, and IKK protein expression. Data represent mean ± SD of six mice; ^#^ *p* < 0.05, ^##^ *p* < 0.01, and ^###^ *p* < 0.001 were compared with sample of control group (one-way ANOVA followed by Scheffe’s multiple range tests); ** *p* < 0.01, and *** *p* < 0.001, were compared with LPS-alone group.

In addition, we also analyzed the expression levels of proteins associated with the TLR4/PI3K/Akt/mTOR/IKKβ signal transduction pathway. As shown in Figure 8A, LPS-induced group exhibited significant increase in TLR4 and PI3K protein expression levels and upregulation of Akt, mTOR, and IKKβ protein expression levels when compared with those of the control group. On the contrary, SS-1-treated group exhibited significantly suppressed activity of the TLR4/PI3K/Akt/mTOR/IKKβ signal transduction pathway when compared with that of the LPS-induced group.

### 2.9. Effects of SS-1 on LPS-Induced Antioxidative Enzymes and HO-1/Nrf2 Protein Expressions in ALI Mice

As shown in Figure 8B, the administration of LPS alone significantly reduced the protein expression levels of GPX, SOD-1, CAT, HO-1, Trx-1, KAP-1, and Nrf2, and significantly increased Keap1 protein expression levels in the lungs when compared with those in the Control group. However, the administration of SS-1 prior to induction significantly upregulated the expression levels of antioxidant enzymes and HO-1/Trx-1 proteins, as well as significantly regulated expression levels of KAP-1/Nrf2 protein in the lung tissue when compared with those in the LPS-induced group.

### 2.10. HPLC Profile of SS-1

**Figure 9:**
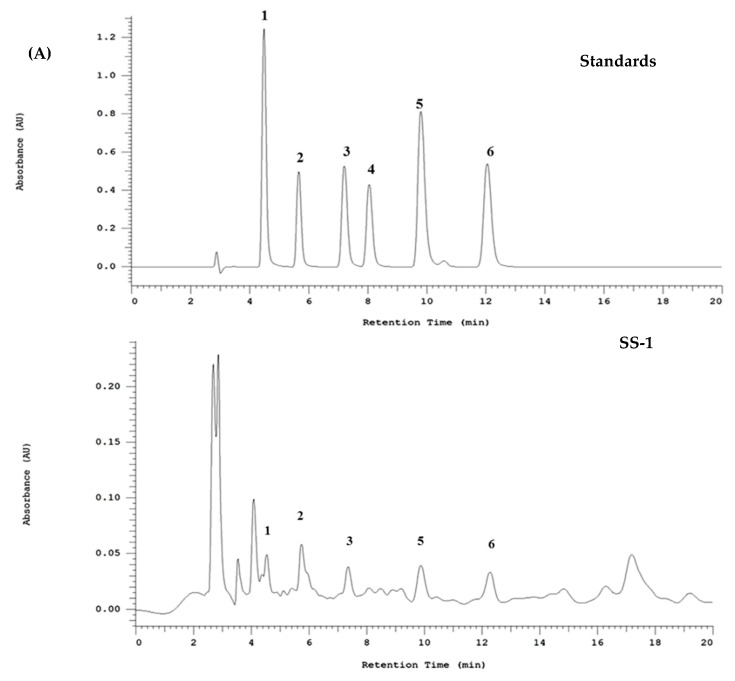

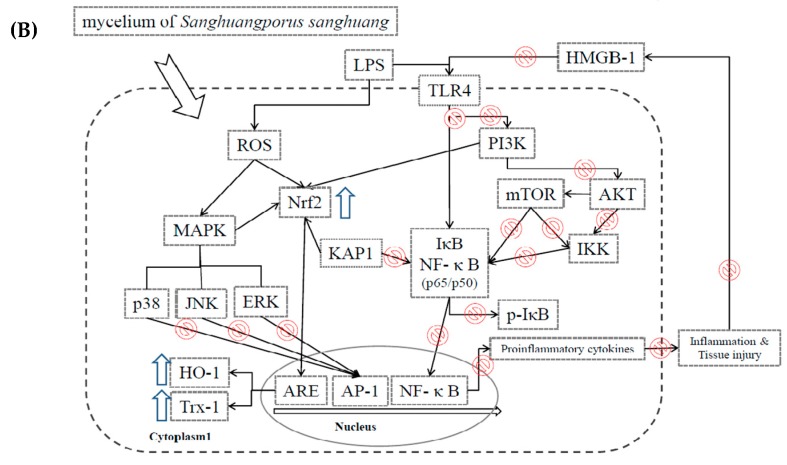
HPLC profile of SS-1 (**A**); and scheme of its mechanism for the protective effect on LPS-induced inflammation (**B**). HPLC chromatogram of the polyphenol standards (500 μg/mL) at 280 nm. Peaks: 1. Protocatechuic acid (4.53 min); 2. Protocatechvaldehyde (5.73 min); 3. Caffeic acid (7.53 min); 4. Syringic acid (8.18 min); 5. DTA (9.89 min); and 6. DBL (12.3 min). Representative chromatograms of SS-1 are shown: 1. Protocatechuic acid (96.8 μg/mg extract); 2. Protocatechvaldehyde (57.2 μg/mg extract); 3. Caffeic acid (59.3 μg/mg extract); 4. Syringic acid (42.6 μg/mg extract); 5. DTA (2,5-dihydroxyterephtalic acid, 80.7 μg/mg extract); and 6. DBL (3,4-dihydroxybenzalacetone, 90.2 μg/mg extract).

Studies have shown that plant polyphenols exhibit great antioxidant capabilities. In addition, they exhibit the ability to capture free radicals and reduce oxidative damage to proteins, lipids and DNA, as well as prevent many diseases caused by the presence of an OH group in its structure. According to the HPLC results presented in this study, we confirmed that the mycelium of *Sanghuangporus sanghuang* is rich in polyphenolic compounds that mainly consist of phenolic acids: Protocatechuic acid (96.8 μg/mg extract), Protocatechvaldehyde (57.2 μg/mg extract), Caffeic acid (59.3 μg/mg extract), Syringic acid (42.6 μg/mg extract), 2,5-dihydroxyterephtalic acid (DTA, 80.7 μg/mg extract), and 3,4-dihydroxybenzalacetone (DBL, 90.2 μg/mg extract), as shown in Figure 9A.

## 3. Discussion

LPS is a cell wall component of Gram-negative bacteria and an endotoxin commonly found in the environment [27]. The intratracheal instillation of LPS is a good method to directly induce ALI in animal models. LPS can induce functional abnormalities in respiratory tract and pulmonary circulation, including changes in blood flow and increased lung tissue permeability, which can cause pulmonary edema, thereby leading to respiratory failure and even death [28,29]. LPS can activate the alveolar macrophages and attract neutrophils to infiltrate the lung tissue at large scales [29,30]. Upon induction by LPS, the neutrophils release free radicals with toxic effects and regulate the synthesis of pro-inflammatory mediators via the NF-κB and MAPK pathways [31].

The results of our cytological experiments showed that the administration of SS-1 at concentrations that do not affect the survival of macrophages, could significantly regulate the expression of genes associated with the LPS-induced inflammatory response and the synthesis of pro-inflammatory substances. SS-1 exhibits good anti-inflammatory capabilities and improves the inflammatory situation of macrophages by inhibiting the activation of the NF-κB signal transduction pathway and the phosphorylation of MAPK. The oral administration of SS-1 at 20, 50, and 100 mg/kg for five consecutive days prior to induction with LPS was found to reduce the wet/dry weight ratio of lung tissue and the total cell count and protein concentration in BALF. We propose that the reason for this effect is that SS-1 can alleviate the pulmonary microvascular and alveolar cell injuries by reducing the pro-inflammatory mediators, thereby altering the pulmonary microvascular permeability. In addition, it may also down-regulate MPO activity in the lung tissue.

The binding of LPS to TLR4 alters the physiological roles of cells by promoting cell survival or activating signal transduction pathways related to the inflammatory response. Previous studies have shown that LPS causes Akt phosphorylation via PI3K and the phosphorylated Akt promotes the activation of NF-κB via IKK [32,33]. Other studies have pointed out that mTOR participates in the regulation of cell survival and the immune response. mTOR is found downstream of the PI3K/Akt signal transduction, Akt phosphorylates mTOR, and the phosphorylated mTOR interacts with IKK to cause the activation of NF-κB. mTOR plays an important role in the LPS-induced inflammatory response, and the inhibition of mTOR reduces the phosphorylation of NF-κB and the expression of pro-inflammatory cytokines in the neutrophils [8,9,10,11,33]. Our experimental results show that the administration of SS-1 prior to induction with LPS can downregulate the signal transduction pathways triggered by the immune response in ALI tissues. Regulation of TLR4 and PI3K protein activation indirectly affects the phosphorylation of Akt, mTOR, and IKK proteins; inhibits the activation and nuclear translocation of NF-κB; and reduces the protein phosphorylation of the MAPK pathway, thereby down-regulating the release of pro-inflammatory cytokines and inflammatory substances from cells to alleviate the injury of lung tissues. In addition, we also found that the administration of SS-1 prior to induction could reduce the concentrations of TNF-α, IL-1β, and IL-6 in BALF due to LPS-induced ALI, as well as increased cytokine synthesis inhibitory factor IL-10 concentration, thus alleviating the lung tissue injury.

HMGB1 has a molecular weight of 30 kDa, and under normal physiological conditions, it is present in the nucleus of all eukaryotic cells to help maintain DNA stability and regulate gene transcription. Recently, an increasing number of studies have shown that HMGB1 plays an important role in inflammatory diseases (including lung and kidney diseases), mainly due to the binding of extracellular HMGB1 to RAGE and TLRs during inflammation to activate the NF-κB signal transduction pathway and produce more pro-inflammatory cytokines and chemokines [7,34,35]. It could also be positively regulated HMGB-1 expression in macrophages can be upregulated to enhance the inflammatory response, and is thus recognized as another type of cytokine [36,37]. Our experimental results show that the administration of SS-1 prior to induction can regulate HMGB1 protein expression levels in the lung tissue with LPS-induced ALI.

Oxidative stress generated by ROS is extensively involved in the physiopathological changes. Tissue and cells have a series of defensive mechanisms in response to oxidative stress and injury to alleviate cell injury [38]. Nrf2 is currently considered a key transcription factor that regulates the defense of cells against oxidative injury [39,40,41,42]. Upon exposure to ROS or the indirect effect of phosphorylation, Nrf2 is released from Keap1 and translocated into the nucleus to bind to the ARE sequence, triggering the transcription of downstream phase II antioxidant genes, such as catalase (CAT), NADPH: quinone oxidoreductase (NQO1), superoxide dismutase (SOD), thioredoxin 1 (Trx-1), and heme oxygenase-1 (HO-1), and enhancing the antioxidant capacity of cells [12,13,43,44]. Present studies suggested that PI3K and MAPK pathways are signal transduction pathways mainly involved in the activation of Nrf2/ARE and its downstream phase II detoxification enzyme, as well as the gene expression of antioxidant enzymes [14,45]. Our experimental results show that the administration of SS-1 prior to induction can reduce the production of ROS in tissues with LPS-induced ALI. SS-1 may regulate the phosphorylation level of proteins related to the MAPK pathway. In addition, SS-1 also regulates the activation of Nrf2 and removes abundant ROS in the tissue with the help of antioxidant enzymes, such as SOD, CAT, GPx-1, HO-1 and Trx-1, thus alleviating the lung tissue injury.

Gene expression in eukaryotic organisms is mainly regulated at the transcriptional level. The auxiliary transcriptional regulator, KAP1 (KRAB-associated protein 1), is present in many transcription complexes and involved in transcription, however, the mechanism of KAP1 in mediating the binding of transcription factors and regulatory proteins is still unclear [46,47]. Previous studies have suggested that KAP1 negatively regulates the signal transduction of the NF-kB pathway and affect the synthesis of pro-inflammatory cytokines (TNF-α and IL-6) by altering the expression levels of STAT3 [16,46,47]. In addition, studies have also shown that KAP1 can positively regulate the signal transduction of the Nrf2 pathway [17,48]. Our experimental results show that the administration of SS-1 prior to induction with LPS can significantly affect the expression levels of KAP1 and indirectly regulate the signal transduction of the NF-kB and Nrf2 pathways, thereby altering the syntheses of pro-inflammatory cytokines and antioxidant enzymes.

*S. sanghuang* has been proven to contain various polyphenolic compounds. Our HLPC analysis confirmed that the mycelium extract of *S. sanghuang* is rich in polyphenolic compounds. As mentioned earlier, the OH groups contained in polyphenolic compounds act as hydrogen donors and, therefore, exert radical scavenging effect. Hence, the extract is considered an important natural source of antioxidants. Recent studies showed that protocatechuic acid exerts a protective effect against LPS-induced acute lung injury in mice by inhibiting the p38MAPK and NF-κB signaling pathways. It has also been reported that caffeic acid exerts anti-inflammatory effect by inhibiting the NF-κB and PI3K/Akt signaling pathways. In addition, current reports pointed out that protocatechualdehyde alleviate oxidative stress-induced injury by activating the Nrf2/HO-1 pathway. On the other hand, two phenolic acids (i.e., 2,5-dihydroxyterephtalic acid and 3,4-dihydroxybenzalacetone) in *Inonotus obliquus*, which is also a medicinal fungus, are believed to eliminate H_2_O_2_-induced oxidative stress. Our experimental results found that SS-1 reduces the production of NO in activated macrophages, with an IC_50_ value of 491.5 μg/mL. Additionally, our previous studies confirmed that protocatechuic acid, protocatechualdehyde, and caffeic acid reduce the production of NO in activated macrophages, with IC50 > 100, 21.4, and 28.2 μg/mL, respectively. Therefore, we speculated that SS-1 exerts protective effect on lung tissues in the animal model of LPS-induced acute lung injury, which may be associated with the anti-inflammatory and antioxidant effects of its polyphenolic compounds that can serve as a source of activity.

Taken together, we can infer from the results of this study that LPS induces ALI and the administration of SS-1 prior to induction protects the tissues against LPS-induced ALI, as shown in Figure 9B.

## 4. Materials and Methods

### 4.1. Source of Material

The mycelium of *Sanghuangporus sanghuang* grown on mulberry tree used in this study was provided by Grape King Bio Ltd. (Taoyuan, Taiwan) and its species was confirmed by Sheng-Hua Wu, a researcher from the Botanical Group of National Museum of Natural Science.

### 4.2. Sample Extraction

Dried powders were immersed in and extracted with 70% ethanol for several days, followed by filtration. Filtrates were concentrated under reduced pressure to remove ethanol. This step was repeated multiple times to obtain the extracts, which were then stored for subsequent analysis.

### 4.3. Cell Culture

A murine macrophage cell line RAW264.7 (BCRC No. 60001) was purchased from the Bioresources Collection and Research Center (BCRC) of the Food Industry Research and Development Institute (Hsinchu, Taiwan). Cells were cultured in plastic dishes containing Dulbecco’s Modified Eagle Medium (DMEM, Sigma, St. Louis, MO, USA) supplemented with 10% fetal bovine serum (FBS, Sigma, St. Louis, MO, USA) in a CO_2_ incubator (5% CO_2_ in air) at 37 °C and subcultured every 3 days at a dilution of 1:5 using 0.05% trypsin 0.02% EDTA in Ca^2+^, Mg^2+^ free phosphate-buffered saline (DPBS).

### 4.4. Cytotoxicity and NO Production

RAW264.7 cells (5 × 10^4^ cells/well) were cultured in 96-well plate in DMEM containing 10% FBS for 24 h to become nearly confluent. Then cells were cultured with increasing concentrations of SS (125–500 μg/mL) in the presence of 100 ng/mL LPS for 24 h. After that, cells were incubated with 100 μL of 0.5 mg/mL MTT (Sigma, St. Louis, MO, USA) for 4 h at 37 °C. After incubation, the colored formazan crystals formed in culture plate was dissolved in 0.04 N HCl/isopropanol. The optical densities (OD) were measured at 570 nm using a microplate reader (Molecular Devices, Sunnyvale, CA, USA). The viability of RAW264.7 cells in each well was presented as compared with percentage of untreated control cells.

NO production was indirectly assessed by measuring the nitrite levels in the culture media using Griess reagent assay. Briefly, RAW264.7 cells were seeded at a density of 5 × 10^4^ cells/well in 96-well plates for 24 h. After incubation, the cells were treated with SS-1 (125, 250 and 500 μg/mL) in the presence of LPS (100 ng/mL) for 24 h. The culture supernatant was collected for nitrite assay. Each of 100 μL of culture media was mixed with an equal volume of Griess reagent (0.1% *N*-1-napthylethylenediamine dihydrochloride in water, 1% sulfanilamide in 5% phosphoricacid) and incubated at room temperature for 5 min, the absorbance was measured at 540 nm with a microplate reader (Molecular Devices). Fresh culture media were used as blanks and the nitrite levels were determined by using a standard curve obtained from sodium nitrite.

Macrophages were seeded at 5 × 10^4^ cells/well in 96-well plates. Cells were incubated with SS-1 (125, 250 and 500 μg/mL) in the presence of LPS (100 ng/mL) for 24 h. Cell culture supernatants were centrifuged at 5000× *g* for 3 min at 4 °C to remove insoluble material. Secreted IL-1β, IL-10, IL-6, and TNF-α were measured in cell culture supernatants using commercially-available ELISA kits (BioLegend, San Diego, CA, USA) following the instructions provided by the manufacturers. The absorbance (450 nm) for each sample was analyzed using microplate reader and was interpolated with a standard curve. Results of three independent experiments were used for statistical analysis.

### 4.5. Animals

Male ICR mice, 6–7 weeks old, were obtained from BioLASCO Taiwan Co., Ltd. (Taipei, Taiwan). The animals were kept in Plexiglas cages at a constant temperature of 22 ± 1 °C, relative humidity 55% ± 5% and with 12 h dark–light cycles. Food and water were given ad libitum.

Animal studies were conducted according to the regulations of Instituted Animal Ethics Committee, and the protocol was approved by the Committee for the Purpose of Control and Super-vision of Experiments on Animals (Protocol No. 2016-376). After a 1–2–week adaptation period, male ICR mice (25–32 g) were randomly assigned to six groups (*n* = 6). The control group received normal saline (intraperitoneal, i.p.). The other groups included LPS-treated (5 mg/kg), positive control (LPS + Dex, 10 mg/kg) and SS-1 administered groups (LPS + SS-1-L, 125 mg/kg; LPS + SS-1-M, 250 mg/kg; and LPS + SS-1-H, 500 mg/kg).

### 4.6. Model of LPS Induced ALI

Seventy-two healthy male ICR mice were randomly divided into 6 groups (*n* = 6): control group, LPS group, dexamethasone (Dex) group (10 mg/kg), low dosage SS-1 group (125 mg/kg), middle dosage group (250 mg/kg, LPS + SS-1-M) and high dosage group (500 mg/kg, LPS + SS-1-H). Take half of each group for the inflammation protein analysis, slicing, and edema and used the rest of the mice for BALF analysis. Mice were intratracheally instilled with 5 mg/kg LPS in 50 μL sterile saline or sterile saline alone (control group). In brief, mice were anesthetized with mixed reagent of 10 μL/g i.p. urethane (0.6 g/mL) and chloral hydrate (0.4 g/mL), followed by Dex (10 mg/kg) or lobeline intraperitoneal injection with individual dose. Six hours later, the mice received sacrifice and bronchoalveolar lavage fluid (BALF) and lung tissues were collected.

### 4.7. Bronchoalveolar Lavage Fluid (BALF), Total Cell Count and Protein Analysis

Six hours later, mice were exsanguinated after anesthesia. According to the previous report, BALF was collected by the upper part of the trachea, by douche three times with 500 μL PBS (pH 7.2). The fluid recovery rate was more than 90%. Lavage sample from each mouse was kept on ice. BALF was centrifuged at 700× *g* for 5 min. The sediment cells were resuspended in 2 mL PBS, half of them have used to detect cell counts by cytometer, the rest equally divided into two parts. One has centrifuged again in order to get sediment for extracting proteins with a RIPA solution (radioimmuno-precipitation assay buffer) and centrifuged again to obtain the supernatant in order to detect total protein content by Bradford assay.

### 4.8. TNF-α, IL-6, and IL-1β Cytokines in BALF

Serum levels of TNF-α, IL-1β, and IL-6 were determined using a commercially available enzyme linked immunosorbent assay (ELISA) kit (Biosource International Inc., Camarillo, CA, USA) according to the manufacturer’s instruction. TNF-α, IL-1β, and IL-6 were determined from a standard curve.

### 4.9. Myeloperoxidase (MPO) Activity Assay

The lungs were homogenized, 12,000× *g* at 4 °C for 15 min and resuspended in 50 mM KPO_4_ buffer (pH 6.0) with containing 0.19 mg/mL of o-dianisidine chloride and 0.0005% H_2_O_2_ was a substrate for myeloperoxidase at 460 nm with a spectrophotometer (Molecular Devices, Sunnyvale, CA, USA). MPO content was expressed as relative MPO activity (OD 460 nm/mg protein of lung tissue).

### 4.10. Lung Wet/Dry Weight Ratio

The lower lobe of the left lung was blotted dry and weighed before being placed in an oven at 50 °C for 48 h to obtain the “dry” weight. The ratio of the wet lung weight to the dry lung weight was calculated to assess tissue edema. The right lungs were used for histopathological examination.

### 4.11. H&E Staining

The right lung was harvested and fixed in 10% buffered formalin for 24 h, dehydrated, embedded in paraffin before being stained with hematoxylin and eosin (H&E) and observed under light microscopy.

### 4.12. Protein Extraction and Western Blot Analysis

RAW264.7 cells were seeded at a density of 5 × 10^6^ cells/dish in 10 cm dish and then with different concentrations of SS-1 (125, 250 and 500 μg/mL) and 100 ng/mL of LPS for 24 h to measure the protein expression levels. The cells were harvested and lysed by RIPA buffer (Thermo Fisher Scientific, Waltham, MA, USA) for 20 min on ice, and the lysates were centrifuged at 10,000× *g* for 15 min at 4 °C.

PBS and RIPA were added to lung tissue before grinding. The extract was then centrifuged at 12,000× *g* for 15 min to obtain the supernatant. Bovine serum albumin (BSA) was used as a protein standard to calculate the equal total cellular protein amounts. Protein samples (50 μg) were resolved by denaturing 10% sodium dodecyl sulfate-polyacrylamide gel electrophoresis (SDS-PAGE) using standard methods, and then were transferred to PVDF membranes (Immobilon, Millipore, Bedford, MA, USA) by electroblotting and blocking with 5% skim milk. The membranes were then incubated with mouse monoclonal anti-iNOS, anti-COX-2, anti-NF-κB (p65), anti-IκB, anti-p-IκB, anti-Nrf2, anti-HO-1, anti-MAPK antibody and antioxidative enzymes (SOD, GPx, Catalase) in TBST at 4 °C overnight, washed three times with TBST, and incubated for 1 h at 37 °C with horseradish peroxidase conjugated secondary antibodies. The membranes were washed three times before being detected for immunoreactive proteins with enhanced chemiluminescence (ECL) using hyperfilm and ECL reagent (Thermo Scientific Hudson, USA). The results of Western blot analysis were quantified by measuring the relative intensity compared to the control by using Kodak Molecular Imaging Software (Version 4.0.5, Eastman Kodak Company, Rochester, NY, USA) and represented in relative intensities.

Antibodies against Nrf2, HO-1, IκB-α, p-IκB-α, NF-κB, P38 and β-actin were obtained from Abcam (Cambridge, UK). Antibodies against iNOS, COX-2, p-ERK1/2, p-JNK, JNK, Trx-1, Keap-1, KAP1, GPX1, CAT, SOD-1, TLR4, AKT and HMGB1 were purchased from Gene Tex (San Antonio, TX, USA). Antibodies against ERK1/2, MPO, IKK, p-IKK and p-mTOR were obtained from Cell Signaling Technology (Danvers, MA, USA). Antibodies against p-P38, p-AKT, and PI3K were purchased from Millipore (Billerica, MA, USA).

### 4.13. Fingerprint Analysis by HPLC

The analysis will be performed on a HITACHI HPLC L-5000 system equipped with adegasser, pumps, and a photodiode array detector linked to a PC computer running the software program HPLC LACHROM. For HPLC analysis, an aliquot (10 μL) is injected into the columns and eluted at 40 °C. The analytical column (250 × 4.6 mm i.d., 5 μm) used is TOSOH TSK-GEL ODS-80T_M_ (Tokyo, Japan), and the detection wavelength. For photodiode array detection, the wavelengths of standard compounds at their respective maximum absorbance wavelength can monitored at the same time. Identification is based on retention times and on-line spectral data in comparison with authentic standards.

The mobile phase contained acidified water with acetic acid (2.5%, solvent A) and Methanol (solvent B). The gradient program started with 10% solvent B for 0 min, then linearly increased to 75% solvent B for another 55 min. This linear gradient was followed by an isocratic elution until 30 min and reconditioning steps to return to the initial mobile phase condition. The flow rate was 0.8 mL/min, and the injection volumes of standards and samples were 10 μL.

### 4.14. Statistical Analysis

Unless otherwise stated, all experiments were performed at least three times independently. Experimental results were presented as the mean ± standard deviation (SD) of three parallel measurements. Statistical evaluation was carried out by one-way analysis of variance (ANOVA) followed by Scheffe’s multiple range tests. Statistical significance was expressed as ^#^ *p* < 0.05, ^##^ *p* < 0.01 and ^###^ *p* < 0.001 were compared with sample of control group; * *p* < 0.05, ** *p* < 0.01, and *** *p* < 0.001, were compared with LPS-alone group.

## 5. Conclusions

Finally, our experimental results showed that mycelium of *S. sanghuang* exhibits good anti-inflammatory properties. Both in vitro and in vivo assays showed that SS-1 can regulate the expression levels of TLR4/PI3K/Akt/mTOR and IKK proteins, inhibit the activation and nuclear translocation of NF-κB, and reduce the phosphorylation of proteins related to the MAPK pathway, thereby reducing the release of pro-inflammatory cytokines and inflammatory substances from cells, improving inflammatory response. In addition, SS-1 could also up-regulate the expression of antioxidant enzymes and HO-1, Trx-1 proteins, inhibit cell inflammation, and remove ROS at large scale by regulating KAP-1/Nrf2 protein expression. The mycelium of *S. sanghuang* has potential in the treatment and/or prevention of inflammatory-related diseases, such as ALI.
